# Harnessing Metabolites as Serum Biomarkers for Liver Graft Pathology Prediction Using Machine Learning

**DOI:** 10.3390/metabo14050254

**Published:** 2024-04-27

**Authors:** Cristina Baciu, Soumita Ghosh, Sara Naimimohasses, Arya Rahmani, Elisa Pasini, Maryam Naghibzadeh, Amirhossein Azhie, Mamatha Bhat

**Affiliations:** Ajmera Transplant Program, University Health Network, Toronto, ON M5G 2C4, Canada; cristina.baciu@uhn.ca (C.B.); soumita.ghosh@uhn.ca (S.G.); sara.naimimohasses@uhn.ca (S.N.); arya.rahmani@uhn.ca (A.R.); elisa.pasini@uhn.ca (E.P.); maryam.naghibzadeh@queensu.ca (M.N.); azhiea@myumanitoba.ca (A.A.)

**Keywords:** metabolomics, liver transplant, T-cell mediated rejection, metabolic dysfunction-associated steatohepatitis (MASH), biliary complications

## Abstract

Graft injury affects over 50% of liver transplant (LT) recipients, but non-invasive biomarkers to diagnose and guide treatment are currently limited. We aimed to develop a biomarker of graft injury by integrating serum metabolomic profiles with clinical variables. Serum from 55 LT recipients with biopsy confirmed metabolic dysfunction-associated steatohepatitis (MASH), T-cell mediated rejection (TCMR) and biliary complications was collected and processed using a combination of LC-MS/MS assay. The metabolomic profiles were integrated with clinical information using a multi-class Machine Learning (ML) classifier. The model’s efficacy was assessed through the Out-of-Bag (OOB) error estimate evaluation. Our ML model yielded an overall accuracy of 79.66% with an OOB estimate of the error rate at 19.75%. The model exhibited a maximum ability to distinguish MASH, with an OOB error estimate of 7.4% compared to 22.2% for biliary and 29.6% for TCMR. The metabolites serine and serotonin emerged as the topmost predictors. When predicting binary outcomes using three models: Biliary (biliary vs. rest), MASH (MASH vs. rest) and TCMR (TCMR vs. rest); the AUCs were 0.882, 0.972 and 0.896, respectively. Our ML tool integrating serum metabolites with clinical variables shows promise as a non-invasive, multi-class serum biomarker of graft pathology.

## 1. Introduction

***Chronic graft injury compromises long-term survival:*** Liver transplantation saves thousands of lives worldwide annually—in fact, there were over 37,000 transplants in 2022, and 13,400 in the Americas alone [1]. However, long-term survival in 25% of liver transplant (LT) recipients is compromised by ongoing graft injury that results in cirrhosis [2]. Liver graft injury is typically signaled by abnormalities in liver biochemistry [3,4]. Causes of graft injury include T-cell mediated rejection (TCMR), metabolic dysfunction-associated steatohepatitis (MASH), biliary complications and viral infections, amongst others [5]. TCMR is the most common cause of liver graft injury, and repeated episodes lead to chronic rejection, premature graft loss and compromised long-term survival [6]. MASH recurs in most patients originally transplanted for MASH (MASH-LT), and an estimated 50% develop significant graft fibrosis (defined as Stage 2 or greater) within 5 years of transplant [7]. Overall, ongoing graft injury leads to accelerated fibrosis in comparison to the native liver, progressing at an estimated rate of 0.4 stages per year, which can rapidly result in cirrhosis and loss of the graft [6,8,9,10,11]. 

***How can we best preserve the long-term health of the liver graft?*** It is imperative that we optimize the long-term outcomes of the graft and its recipient using a personalized, data-driven approach. The only way to reliably diagnose graft injury at present is by performing a liver biopsy and assessing histological features. However, a liver biopsy is an invasive procedure with a 1.8% risk of complications, and it is impractical to perform longitudinal liver biopsies over a LT recipient’s lifetime [12]. Circulating cell-free DNA (cfDNA) has been investigated as a potential non-invasive biomarker of TCMR [13]. A significant rise in cfDNA can be used to differentiate TCMR from normal graft function and non-TCMR graft injury with Area Under Curve (AUC) of 0.95 and 0.71, respectively [14]. However, cfDNA is unable to differentiate between different graft pathologies. Levitsky et al. used a panel of gene expression signatures to distinguish LT recipients with TCMR from another group encompassing all other graft pathologies (AUC = 0.83, accuracy 0.78, sensitivity 0.70, specificity 0.81) [15]. Therefore, there is a great need for effective serological biomarkers to facilitate the noninvasive diagnosis of graft pathologies, such as TCMR, biliary complications and MASH.

The term ‘metabolomics’ describes the identification and quantification of metabolites in biological tissue [16]. Metabolites, as downstream products of gene expression, protein and enzymatic function, can provide valuable information on the biological processes within a cell, tissue, organ system or organism, in addition to the pathophysiology behind different disease states [16,17]. In a pre-transplant population, previous studies have identified multiple lipid and amino acid serum metabolites associated with various liver pathologies, including advanced MASH [18,19,20,21]. However, the data are quite limited, especially in post-transplant populations, although early single center studies have identified potential metabolomic profiles in donors associated with early allograft dysfunction, indicators of ischemia-reperfusion injury in recipients and TCMR in pediatric populations [17,22,23].

This study aimed to identify distinct metabolomic profiles in the serum of individuals following liver transplant, with the goal of recognizing potential biomarkers capable of differentiating between post-transplant complications, specifically MASH, TCMR, and biliary issues. Serum metabolomics is relatively cost-effective as compared to other high-throughput approaches. We employed a random forest (RF) algorithm to develop a classification model distinguishing between biliary complications, MASH, and TCMR. The interpretation of the model was conducted through the permutation-based feature importance measurement for random forests [24].

## 2. Materials and Methods

### 2.1. Sample Collection and Processing

Serum samples from consented patients with MASH (n = 10), TCMR (n = 18), biliary complications (n = 27) were retrieved from the Multi Organ Transplant Program at the Ajmera Transplant Centre (UHN). Serum samples were processed for targeted metabolomics processing by The Metabolomics Innovation Centre (TMIC, Edmonton, AB, Canada, https://metabolomicscentre.ca) using a combination of direct injection mass spectrometry with a reverse-phase LC-MS/MS custom assay. This specialized assay enabled the accurate identification and quantification of 143 native metabolites encompassing amino acids, acyl carnitines, biogenic amines and their derivatives, uremic toxins, glycerophospholipids, sphingolipids, as well as various sugars. Mass spectrometric analysis was performed on an ABSciex 4000 Qtrap^®^ tandem mass spectrometry instrument (Applied Biosystems/MDS Analytical Technologies, Foster City, CA, USA) equipped with an Agilent 1260 or Waters series UHPLC system. The samples were delivered to the mass spectrometer by an LC method followed by a direct injection (DI) method. The mass spectrometer was set to a positive electrospray ionization mode with a scheduled multiple reaction monitoring (MRM) scan. The Ion Spray voltage was set at 5500 volts and the temperature at 500 °C. The curtain gas (CUR), ion source gas 1 (GAS1), ion source gas 2 (GAS2) and collision gas (CAD) were set at 20, 40, 50 and medium, respectively. More details of the methodology were added to the Supplementary Materials.

### 2.2. Data Analysis

Metabolite concentrations reported in μM units (absolute concentrations) for each sample were used as input for MetaboAnalyst 5.0 software [25] for bioinformatics analysis. For each two-group comparison, the data underwent a series of processing steps, including: (i) removing features with more than 50% data missing, (ii) missing value imputation by replacing missing values with 1/5 of the minimum positive value, and (iii) normalization using quantile normalization, log10 transformation, and autoscaling. Subsequently, we applied multivariate analysis with the Partial Least Square Discriminant Analysis (PLS-DA) approach to identify significant metabolites based on the Variable Importance in Projection (VIP) score calculated for each component. A metabolite was considered significant if VIP > 1. Further, for each of the three outcomes, we performed sex-based stratified analysis using PLS-DA. These metabolites were then categorized into their respective compound classes as per the Kyoto Encyclopedia of Genes and Genomes (KEGG) and the Human Metabolome Database (HMDB) using the Pathway Analysis module of the software. Finally, we individually mapped them onto their primary biochemical pathways to gain a comprehensive visual representation of the metabolic changes. Boxplots illustrating the normalized expression of the significant metabolites and associated *p*-values from unpaired *t*-tests were generated using GraphPad Prism V.10.1.0 (GraphPad Software, San Diego, CA, USA).

### 2.3. Integration of Clinical Variables with VIP Metabolites for Prediction of Patient Outcomes Post Transplantation 

In this study, the cohort of 55 patients was divided into a train–test strategy with a split ratio of 75–25. Subsequently, on the training dataset, a feature selection step was performed to identify key metabolites exhibiting differential abundances across the three classes. We performed receiver operating characteristic (ROC) curve analysis using the filterVarImp function from the R package ‘caret’ [26]. Each metabolite underwent univariate evaluation, and three pairwise comparisons were conducted (Biliary vs. MASH, Biliary vs. TCMR, and MASH vs. TCMR). The maximum area under the curve (AUC) was recorded for each pairwise comparison. Only metabolites surpassing an AUC threshold > 0.75 in at least two pairwise comparisons were considered significant. This feature selection procedure identified a subset of 20 metabolites deemed crucial for the classification task.

Subsequently, we integrated the metabolomic profiles with clinical and laboratory measurements of individuals to train a 3-way Random Forest classifier using the randomForest package [27] in R. Before model training, we normalized both the metabolomic profiles and clinical variables. To address class imbalances, particularly in the minority MASH and TCMR classes, and achieve a balanced class distribution, the Synthetic Minority Over-Sampling Technique (SMOTE) method [28] was employed to generate synthetic samples. The quality of samples generated using SMOTE was visually assessed through t-distributed stochastic neighbor embedding (t-SNE) [29] projections.

The efficacy of the three-class Random Forest classifier was assessed through the evaluation of the Out-of-Bag (OOB) error estimate. To further elucidate the multi-class classification problem, we employed a One-vs-Rest strategy, breaking it down into three distinct binary problems: Biliary versus Rest, MASH versus Rest, and TCMR versus Rest. Mitigating class imbalances in the binary outcomes involved under sampling the majority class. Further, we computed Area Under the Curve (AUC) values to gauge the effectiveness of each model in distinguishing between classes, leveraging a combination of metabolites and clinical markers.

To assess the significance of each explanatory variable (both metabolite and clinical variable), we employed a permutation-based variable-importance measure and Gini impurity criterion, which evaluated the capacity of predictors to mitigate data impurity or disorder. This assessment was conducted using Out-of-Bag data from the RF models.

## 3. Results

A total of 55 participants were enrolled in the study, of which 10 had a diagnosis of post-LT MASH, 18 had TCMR, and 27 had biliary obstruction (Table 1). Most study participants were transplanted for steatotic liver disease, none of our patients had features of cirrhosis on ultrasound, and there was no significant demographic inter-group variability. Expectedly, alanine aminotransferase (ALT) levels were higher in participants with TCMR and MASH whilst alkaline phosphatase (ALP) levels were more elevated in individuals with biliary obstruction.

Within each two-group comparison, a total of 132 metabolites successfully passed the MetaboAnalyst processing steps detailed in the Materials and Methods section. Following these steps, the normalization was applied for further univariate and multivariate analysis.

### 3.1. Alanine, Aspartate, and Glutamate Metabolism Pathway Exhibited Notable Alterations in a Comparative Analysis of MASH (n = 10) and TCMR (n = 18) Patients 

**Figure 1:**
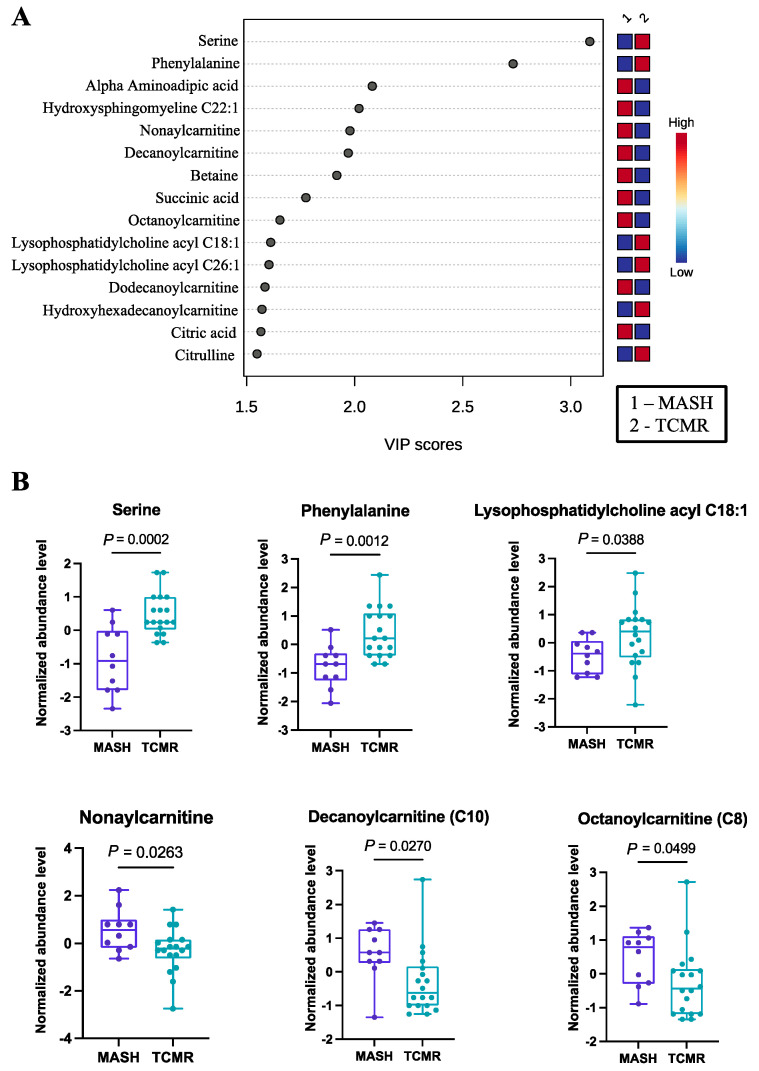
Significant metabolites by MASH vs. TCMR comparison. (**A**). PLS-DA plot with top 15 VIP metabolites. (**B**)**.** Boxplots of selected metabolites. *p*-values were computed by unpaired *t*-tests applied to normalized data.

Employing PLS-DA, we identified 40 important metabolites, as documented in Table S1. The PLS-DA plot, as well as the top 15 features, are graphically depicted in Figure 1A. Among these significant metabolites, several amino acids (serine, phenylalanine, alpha aminoadipic acid) and cholines (lysophosphatidylcholine acyl C18:1, lysophosphatidylcholine acyl C26:1) exhibited higher abundance in TCMR patients. Each of these amino acids have either been implicated in immune modulation or as biomarkers of steatohepatitis, in some cases both [30,31,32,33]. Serine deficiency, for example, has been repeatedly identified as a biomarker of MASH in non-transplant populations, and seems to also have a well-defined role in T-cell responses [34].

The carnitines (nonaylcarnitine, decanoylcarnitine, octanoylcarnitine, dodecanoylcarnitine) were significantly more prevalent in the MASH group. Selected metabolites with their normalized concentrations are illustrated in Figure 1B.

Conducting pathway analysis with VIP metabolites unveiled three significantly perturbed signaling pathways. Among these, the alanine, aspartate, and glutamate metabolism pathway was the most significantly affected, with an FDR-adjusted *p*-value of 1.09 × 10^−3^ (Table 2).

### 3.2. Aminoacyl-tRNA Biosynthesis Emerged as the Pathway Exhibiting the Most Pronounced Alterations When Comparing MASH with Biliary Complications Group 

**Figure 2:**
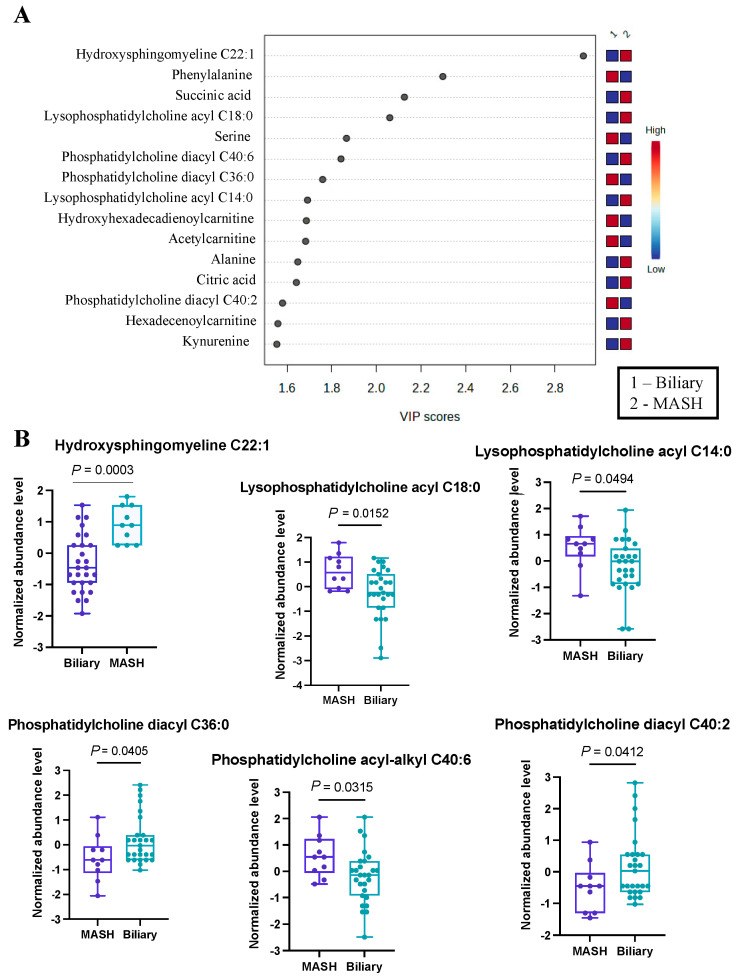
Significant metabolites by MASH vs. Biliary group comparison. (**A**)**.** PLS-DA plot with top 15 VIP metabolites. (**B**)**.** Boxplots of selected metabolites. *p*-values were computed by unpaired *t*-tests applied to normalized data.

As a secondary analysis, we undertook a comparison between MASH patients (n = 10) and those with biliary complications (n = 27). The application of Partial Least Squares Discriminant Analysis provided valuable insights, particularly through the visualization in the PLS-DA plot, which demonstrated a clear and effective separation of samples based on the respective diseases. To offer a more comprehensive understanding of the results, we have presented the top 15 metabolites in Figure 2A.

From this analysis, we identified 39 metabolites with VIP scores greater than or equal to 1, and these findings are detailed in Table S2. Our results indicated hydroxysphingomyeline C22:1 as the top changed metabolite (Figure 2B), with VIP > 2.9, most prevalent in MASH patients. Also, several phosphatidylcholines (lysophosphatidyl acyl cholines C14:0 and C18:0) and phosphatidylcholine acyl-alkyl C40:6 were more abundant in the MASH group. On the contrary, a two phosphatidyldiacyl cholines, C36:0 and C40:2, were found to be more prevalent in patients with biliary complications. Additionally, we noted an elevated abundance of serine and phenylalanine within this group. This differential metabolite distribution underscores the distinctive metabolic profiles associated with these distinct disease conditions. While some of these metabolites have not previously been identified in the development of either complication, a theoretical mechanism exists to explain their abundance or scarcity. For example, hydroxysphingomyeline C22:1 is a ceramide. Ceramide metabolism has been previously identified as being highly upregulated in non-transplant patients with MASH, supporting its potential as an effective biomarker in transplant populations [35].

Using the top 39 metabolites, we identified the three most significant metabolic pathways (Table 2), of which Aminoacyl-tRNA biosynthesis is the most significantly altered (FDR < 1.35 × 10^−6^), with the contribution of eight metabolites, mostly amino acids (Asparagine, Phenylalanine, Glycine, Aspartate, Valine, Alanine, Isoleucine, Leucine, Tryptophan) from our list.

### 3.3. Significant Alterations in Butanoate (Butyrate) Metabolism Were Revealed in the Biliary vs. TCMR Group Comparison 

**Figure 3:**
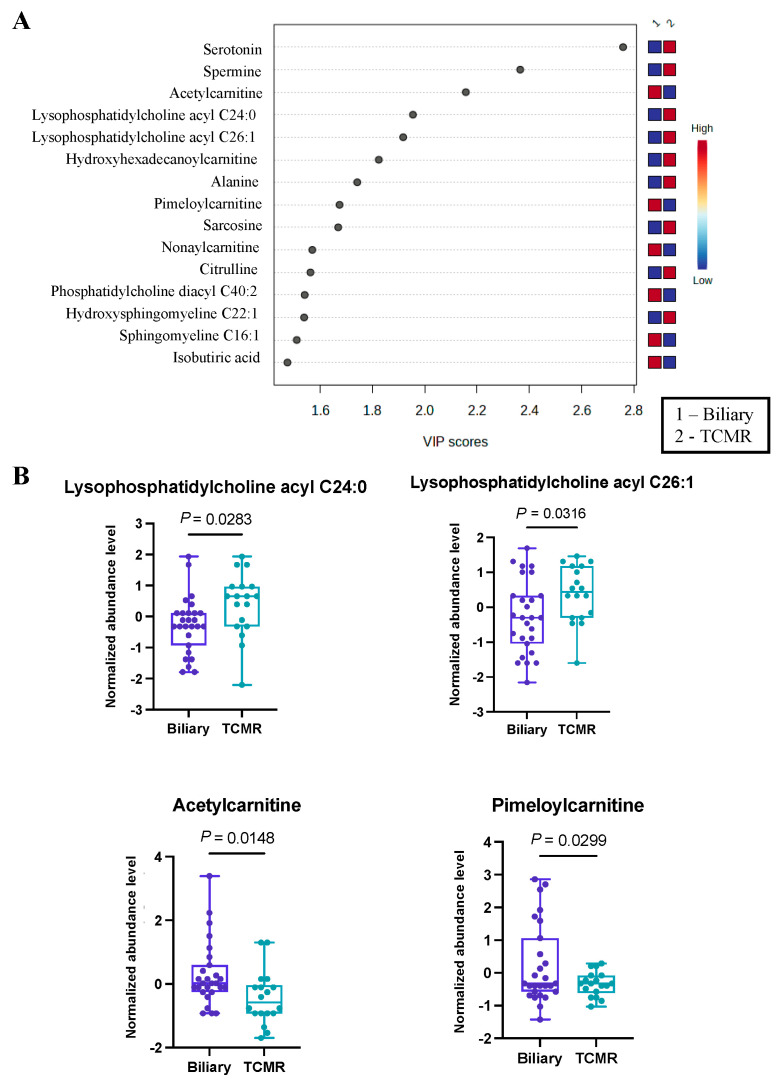
Significant metabolites by TCMR vs. Biliary group comparison. (**A**). PLS-DA plot with top 15 VIP metabolites. (**B**). Boxplots of selected metabolites. *p*-values were computed by unpaired *t*-tests applied to normalized data.

The third and final comparative analysis was performed between individuals with biliary complications and those in the TCMR group. A shorter list of just 28 significant metabolites (Table S3) was revealed by PLS-DA, with the top 15 being illustrated in Figure 3A. Within this set of metabolites, it was evident that serotonin and a pair of lysophosphatidyl acyl cholines (specifically C24:0 and C26:1) exhibited greater abundance in the TCMR group. In contrast, a couple of carnitines, specifically acetyl carnitine and pimeloyl carnitine, were more prevalent among patients with biliary complications (Figure 3B). While serotonin has not previously been identified as a biomarker of post-LT complications, there is a strong biological mechanism for its marked difference in biliary vs. TCMR groups, as serotonin is a potent modulator of T cells and has multiple functions in the liver, including regulation of the biliary tree and cholangiocytes [36,37,38].

Furthermore, employing pathway analysis on metabolites with VIP scores exceeding 1, we identified three significantly altered metabolic pathways, as presented in Table 2. Among these pathways, the most pronounced alterations were observed in the butanoate (butyrate) metabolism pathway, characterized by a false discovery rate value of less than 8.13 × 10^−4^.

In summary, the metabolomics analysis alone unveiled disease-specific metabolic alterations and highlighted the unique biological relevance of certain metabolites within these distinct pathways for each disease condition.

### 3.4. Carnitines Were Identified as Differentially Abundant in Our Disease-Wise Sex-Based Analysis

The inherent metabolic differences between men and women are well known [39], which prompted us to stratify our disease groups by sex. However, our analysis is limited by fewer female participants (n = 5) in MASH and TCMR groups. We applied PLS-DA for our comparative analyses, similar to the previous sections. Metabolites with VIP score > 1 were considered significant in delineating differences between males and females in each group. An overview of the PLS-DA score shows a very good distinction between the sexes (Supplementary Figures S2–S4, A panels) within MASH, TCMR or biliary groups. Several carnitines were identified among the top 15 significant metabolites distinguishing between male and female patients across all disease groups. For instance, in the MASH group, we detected higher levels of propionylcarnitine, hydroxyoctadecenoylcarnitine and carnitine in male patients (Supplementary Figure S2B). Similarly, higher levels of tiglylcarnitine, butyrylcarnitine, propionylcarnitine and valerylcarnitine were found in male versus female patients within the TCMR group (Supplementary Figure S3B). In the biliary group, where we found the highest number of significant carnitines separating males and females, the butyrylcarnitine, malonylcarnitine, hexenoylcarnitine, hydroxyoctadecenoylcarnitine, hydroxytetradecadienoylcarnitine, octadecadienyl-carnitine and methylglutarylcarnitine were more abundant in female patients (Supplementary Figure S4B). Previous studies have shown that carnitines are involved in beta-oxidation of fatty acids in mitochondria [40]. However, further in-depth analysis with increased sample size is needed to elucidate any potential sex-specific differences in carnitine metabolism and its implications.

### 3.5. Integration of Clinical Information with Metabolomics Data

Prior to integration with conventional clinical and laboratory measurements, we performed feature selection on the metabolomics data using ROC curve analysis to retain only the most discriminative metabolites. Employing a random train–test split of 75–25% samples, metabolite concentrations from the training set (n = 40) were utilized to compute AUCs for each pairwise comparison, resulting in the elimination of 112 metabolites. Twenty metabolites were identified as crucial predictors, with AUC thresholds exceeding 0.75 for at least two of the classes. Notably, decanoylcarnitine C10, citric acid, succinic acid, phenylalanine, and serine emerged as the top-ranking metabolites, and their details are presented in Table 3.

Subsequently, we developed a Random Forest classifier model by combining the eight key clinical variables: age, sex, alkaline phosphatase (ALP), alanine transaminase (ALT), aspartate aminotransferase (AST), creatinine, hemoglobin (HGB), and primary indication for transplantation, integrated with the top 20 selected metabolites. The t-SNE projections of the original dataset are shown in Figure S1A. The SMOTE-generated samples have good coherence with the original samples as shown in Figure S1B. A three-way classification was performed to predict the likelihood of a patient belonging to one of three classes: Biliary, MASH, or TCMR. Model parameters were tuned using the Out-of-Bag (OOB) error, revealing 5 as the optimal number of candidate predictors randomly drawn for a split and the number of trees as 500, based on minimum OOB error. The three-class classification model yielded an overall OOB estimate of the error rate at 19.75%.

**Figure 4:**
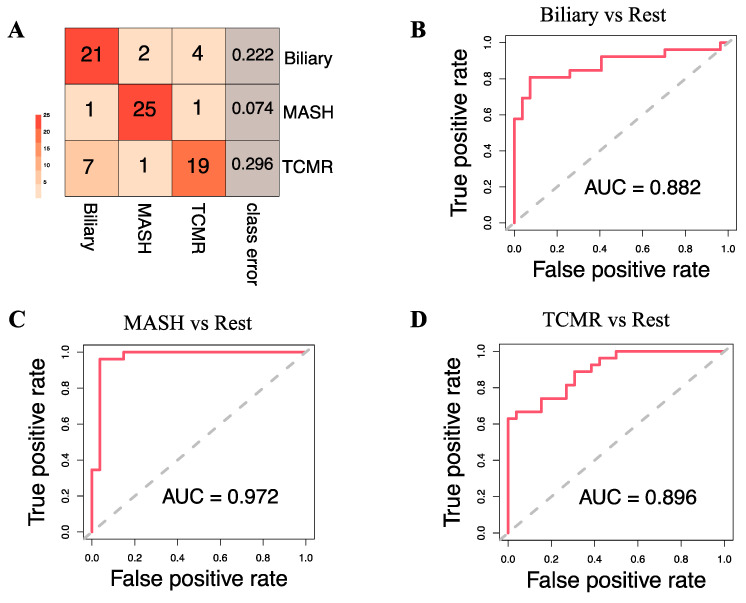
Model Evaluation. (**A**). Classification results on the Out-of-Bag (OOB) samples. The OOB estimate of the error rate is 19.75% for the 3-way classification model. (**B**–**D**) show the Receiver Operating Characteristic (ROC) curve and corresponding Area Under the Curve (AUC) statistics for the following binary models: Biliary vs. Rest, MASH vs. Rest, and TCMR vs. Rest, respectively.

Figure 4A illustrates that at the individual class level, the model demonstrated the maximum ability to distinguish MASH samples with an OOB error estimate of only 7.4%, compared to 22.2% and 29.6% for Biliary and TCMR groups, respectively. The three-way classifier model achieves an overall accuracy of 79.66%. Serotonin and serine have surfaced as the primary predictors, identified through both Mean Decrease in Accuracy and assessment via the Gini Impurity Criterion, which gauges the capacity of predictors to mitigate data impurity or disorder.

Apart from the three-way classification, we also evaluated three Random Forest models predicting binary outcomes: Biliary model (Biliary vs. Rest), MASH model (MASH vs. Rest), and TCMR model (TCMR vs. Rest). The resulting OOB error rates were found to be 22.64%, 5.66%, and 24.53% for the Biliary, MASH, and TCMR models, respectively. Figure 4B–D show the ROC curves and the rank of the variables for the three models. The AUCs for the Biliary, MASH, and TCMR models are 0.882, 0.972, and 0.896, respectively.

**Figure 5:**
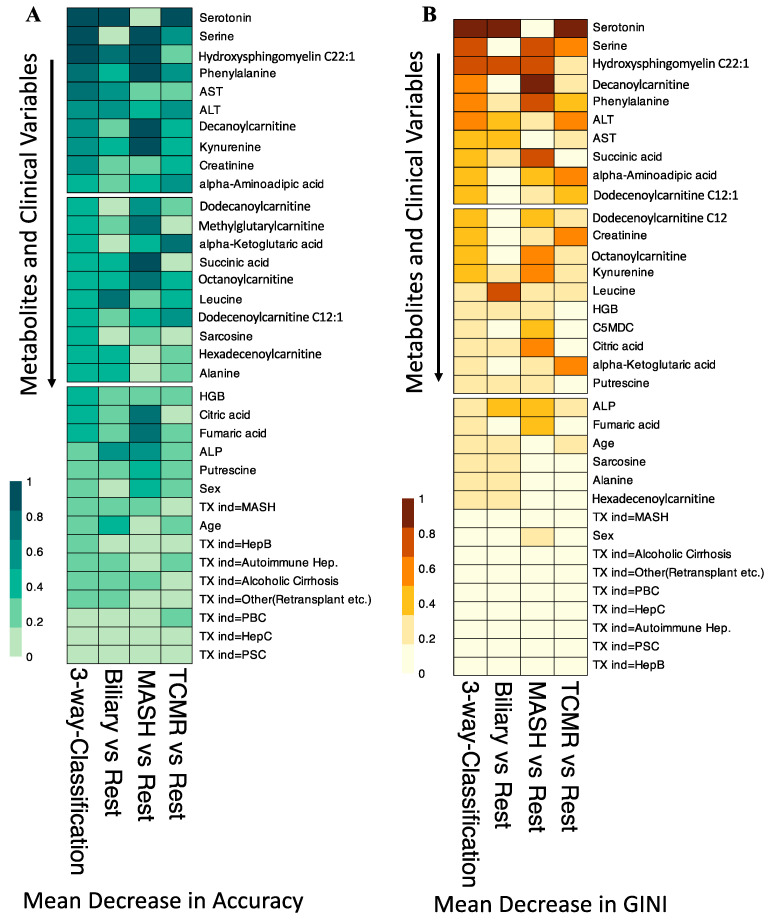
Variable Importance for the Random Forest Classification Models. (**A**). Shows the scaled Mean Decrease in Accuracy over all Out-of-Bag (OOB) cross-validated predictions. The drop in prediction performance for the 3-way classification, Biliary vs. Rest, and TCMR vs. Rest is highest when the metabolite serotonin is omitted. (**B**). The scaled GINI index, a measure of node impurity. Serotonin has the highest GINI index and is again considered the most important variable to split the data correctly by the 3-way classification, Biliary vs. Rest, and TCMR vs. Rest models. TX ind = primary indication for transplant.

In our variable importance analysis, as shown in Figure 5, serotonin emerged as a top predictor for the three-way classification, Biliary, and TCMR models, consistent with Mean Decrease in Accuracy and Mean Decrease in GINI.

**Figure 6:**
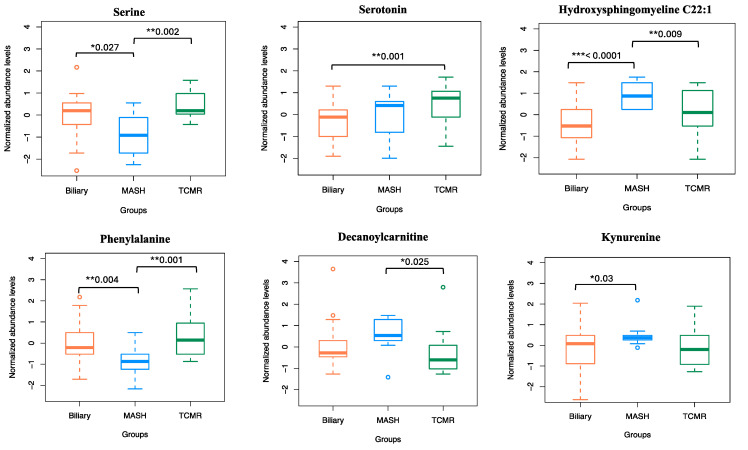
Normalized abundance levels of top predictor metabolites. Shows the normalized abundance levels of top six predictor metabolites across sample groups. The *p*-value used to signify the difference in expression levels among the three groups are computed using independent *t*-test. Only the significant *p*-values are shown.

The amino acid serine and hydroxysphingomyeline (SM(OH)C22:1) emerged as leading predictors for 3-way classification and the MASH model, indicating their importance in classifying MASH samples. Additionally, phenylalanine, decanoylcarnitine, and kynurenine were crucial predictors of MASH. The liver enzymes AST and ALT appeared as the topmost important clinical variables. Abundance levels of top predictor metabolites are plotted in Figure 6.

### 3.6. Comparative Analysis of Integrated Model versus Individual Modalities

**Figure 7:**
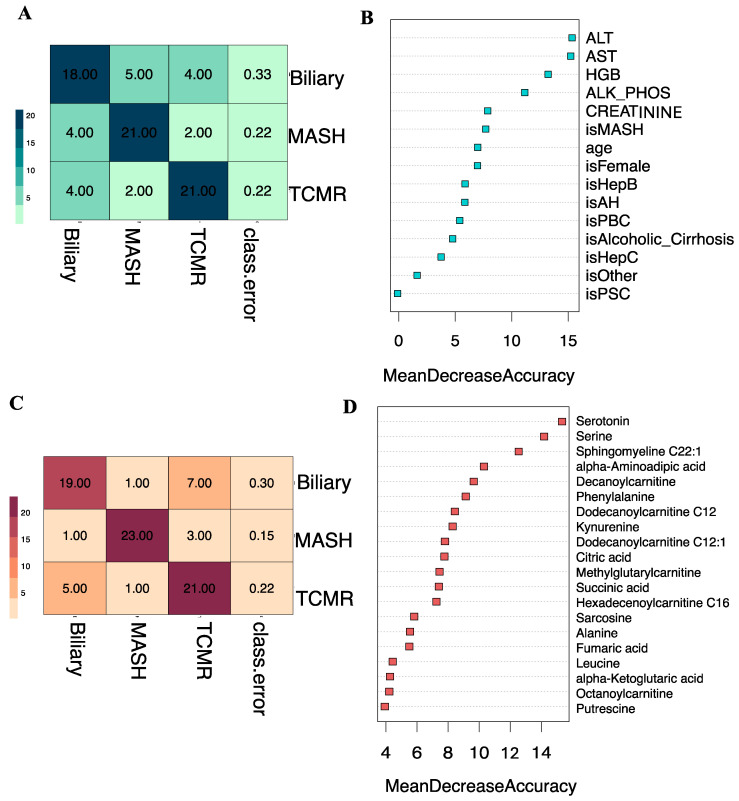
Clinical-only and Metabolite-only models. (**A**). Classification results of clinical-only model on the Out-of-Bag (OOB) samples. The OOB estimate of the error rate is 25.93% for the 3-way classification model. (**B**). Shows the Mean Decrease in Accuracy over all Out-of-Bag (OOB) cross-validated predictions for the clinical-only model. (**C**). Classification results of metabolite-only model on the Out-of-Bag (OOB) samples. The OOB estimate of the error rate is 22.22% for the 3-way classification model. (**D**). Shows the Mean Decrease in Accuracy over all Out-of-Bag (OOB) cross-validated predictions for the metabolite-only model.

We additionally assessed our integrated three-way classification model, which combines clinical variables and metabolites, alongside two other three-way classification models trained solely on a single data type: (i) clinical variables alone, and (ii) metabolites alone. Our Random Forest classifier, when trained on solely clinical variables, produced an overall OOB estimate of the error rate at 25.93%. At the individual class level, depicted in Figure 7A, the model distinguished both MASH and TCMR groups, with OOB error estimates of 22.2% and 33.3% for the Biliary group. The three-way clinical-only model achieves an overall accuracy of 73.37%. The top clinical predictors were ALT and AST, as shown in Figure 7B. While these conventional clinical variables can indicate liver graft injury, they are not specific to the etiologies of graft pathology.

In contrast, our Random Forest classifier trained on top ranked metabolites alone, produced a lower overall OOB estimate of the error rate at 22.22%. Depicted in Figure 7C, the confusion matrix for the metabolites-only model illustrated improved performance at individual class levels, particularly evident in the MASH group with an OOB error estimate of 14.81% compared to the clinical-only model. Achieving an overall accuracy of 77.14%, the metabolite-only model identified serine and serotonin as top predictor metabolites, based on the mean decrease in accuracy, as shown in Figure 7D.

## 4. Discussion

In this study, we aimed to identify distinct, measurable metabolomic profiles to differentiate causes of liver graft injury, along with clinical variables in post-transplant populations. Our metabolomic analysis detected specific metabolites that exhibited significant changes in individuals with TCMR, biliary complications and MASH post-liver transplant. In addition to identifying individual metabolites that varied between disease groups, pathway analysis was conducted to determine which metabolic pathways were most differentially affected.

In the case of MASH vs. TCMR, amino acid metabolism was broadly highlighted, with two of the main pathways affected being alanine aspartate glutamate metabolism and arginine biosynthesis. Both pathways have been affected in steatohepatitis in non-transplant populations and in rejection for transplant populations, further supporting their potential inclusion as relevant biomarkers in post-LT complications [41,42,43].

Comparing MASH vs. Biliary groups, amino acid metabolism was again significant, with branch chain AA(BCAA) synthesis and alanine aspartate glutamate metabolism being highlighted. The BCAA synthesis pathway may be particularly useful as a biomarker of MASH, as levels of these amino acids have been shown to increase greatly in non-transplant MASH populations [44].

Lastly, the biliary vs. TCMR groups highlighted one unique metabolic pathway, butanoate metabolism. Butanoate is a short chain fatty acid synthesized by enteric bacteria and has been repeatedly implicated in mediating tolerogenic phenotypes of T cells, and may therefore be associated with graft tolerance or rejection [45,46]. Considering its unique role in mediating immune functioning, biomarkers associated with butanoate metabolism may be effective in identifying TCMR.

We then developed an ML tool, a Random Forest classifier to predict liver graft pathology, and compared its accuracy on three circumstances: when using only (i) clinical parameters, (ii) metabolites, and (iii) integrating both categories. The improvement in overall accuracy observed in the integrated model, as discussed in Section 3.5, reaffirms our assertion that the complete complexity of graft pathology cannot be adequately captured by any singular data modality. Our results show that clinical variables and metabolites are complementary in nature, and integrating them provides a more comprehensive understanding of liver graft pathology.

When integrating metabolomic data with clinical information via ML modeling, serotonin was identified as a top predictor for the three-way classification. Serotonin has been shown to regulate fibrosis progression [47,48], with higher levels being linked to increased chances of developing MASLD [49]. Our ML model also revealed serine and hydroxysphingomyeline (SM(OH)C22:1) as key metabolites for 3-way classification and the MASH model. Indeed, previous studies have shown the association between serine deficiency and MASH [40,50]. Sphingomyelin is the most frequently observed sphingolipid in mammalian cells, and circulating levels have shown promise as a noninvasive biomarker of MASH [50]. It is considered a bioactive lipid, functioning as a component of cell membranes, with a role in cell signaling, growth, death, senescence, adhesion and migration. The liver plays an important role in lipid metabolism, taking up free fatty acids, forming triglycerides and VLDL in addition to acting as a site for lipid storage. Lovric et al. identified a positive correlation between serum concentrations of sphingomyelin with increased ectopic fat accumulation (including hepatic steatosis) [51,52]. In murine models, it has been shown that sphingomyeline synthesis is activated in MASH, being related to hepatocyte pyroptosis [53]. However, Zhou et al. noted a significant decrease in serum sphingomyelin and lysophosphatidylcholine levels in individuals with MASH versus MASLD [54].

Other top metabolites as predictors for MASH were phenylalanine, decanoylcarnitine, and kynurenine. In agreement with our results, other studies have indicated an increased level of the phenylalanine in MASH patients [55]. Hanssen et al. have shown alteration of the kynurenine pathway in MASLD patients, favoring inflammation and fibrosis through regulation by inflammation markers such as IFN, IL6, LPS [56].

Interestingly, our results showed that carnitines were more prevalent in MASH. Studies looking at the different diets on the serum metabolomic profile noted an increase in both long- and short-chain acyl carnitines with a traditionally more obeso-genic, ‘Western’ diet when compared to individuals on a vegetarian diet and associated with increased risk of cardiovascular disease [47,48]. Additionally, there was a positive correlation between short chain acyl carnitines and fasting insulin levels [47]. Under normal physiologic conditions, carnitine plays an important role in the ß oxidation of fatty acids by facilitating the transmembrane transfer of acetyl-CoA via acetyl carnitine. In pathological situations, including obesity and MASH, where there is an excess of fatty acids due to insulin resistance, increased lipogenesis and impaired fatty acid oxidation, acyl carnitine levels have been shown to accumulate [40,50], with one study demonstrating an AUC > 0.90 for MASH with elevated levels of long chain acyl carnitines: C20, C16:1 and C14:1OH, in the pre-transplant population [50].

A recent review summarizes non-invasive biomarkers in liver transplantation [57] and their performances in Table 1 and Table 2 within. Although gene, protein, and immune cell markers have been extensively researched, only one metabolomic study has previously compared recurrent MASLD patients with those with normal liver function and acute rejection, identifying 14 altered metabolites specific to recurrent MASLD [42]. Our study extensively assesses the diagnostic potential of metabolomic biomarkers in a post-LT cohort. Using our ML modeling approach, we have integrated metabolites with routinely available clinical variables, showcasing high diagnostic accuracies for the three graft pathologies: Biliary (AUC = 0.882), MASH (AUC = 0.972), and TCMR (AUC = 0.896). 

## 5. Conclusions

In this project, we have developed an ML tool integrating serum metabolites with clinical variables in liver transplant patients with MASH, TCMR and biliary complications. Our tool appears to be a promising non-invasive indicator for detecting graft pathology. The model identified serine and serotonin as top altered metabolites, and liver enzymes AST and ALT as the most important clinical variables. It also exceled in predicting the occurrence of MASH following a transplant with the highest accuracy, with an OOB error estimate of 7.4% compared to 22.2% for biliary and 29.6% for TCMR. In the prediction of binary outcomes across three models: Biliary (biliary vs. others), MASH (MASH vs. others), and TCMR (TCMR vs. others), the Area Under the Curve (AUC) scores were 0.882, 0.972, and 0.896, respectively. 

As a limitation to note, our dataset accurately reflects the group of patients from our own institution, without incorporating data from an external cohort for validation purposes. Despite this limitation, it is important to recognize that this work serves as a pilot study featuring a thoroughly detailed and carefully selected group of patients. The outcomes from this study are promising, highlighting the potential of our approach.

## Figures and Tables

**Table 1:** Liver transplant (LT) recipient clinical and laboratory characteristics.

Variable	MASH (n = 10)	TCMR (n = 18)	Biliary Obstruction (n = 27)	*p*-Value
**Recipient age at LT** (years)	54.5 [47,67]	52 [46,60]	59 [53,67]	0.246 ^a^
**Sex**				0.489 ^b^
M	5 (50%)	13 (72.2%)	18 (66.7%)	
F	5 (50%)	5 (27.8%)	9 (33.3%)	
**Laboratory readings** (U/L)				
ALT	77 [45,165]	115 [83,305]	55 [37,90]	0.006 ^a^
AST	45.5 [28,132]	76 [47,198]	36 [25,43]	0.001 ^a^
ALP	168.0 [104,684]	205 [122,329]	241 [147,408]	0.478 ^a^
Creatinine	98.5 [71,140]	80 [71,107]	100 [81,131]	0.546 ^a^
Hgb	123.5 [85,146]	112 [105,120]	104 [94,115]	0.161 ^a^
**Indication for transplant**				0.837 ^b^
ArLD	4 (40%)	4 (22.2%)	9 (33.4%)	
MASH	3 (30%)	2 (11.1%)	4 (14.8%)	
HBV	1(10%)	2 (11.1%)	1 (3.7%)	
HCV	-	3 (16.7%)	3 (11.1%)	
PBC	-	2 (11.1%)	1 (3.7%)	
PSC	-	1 (5.6%)	2 (7.4%)	
Autoimmune hepatitis	-	-	3 (11.1%)	
Other	2 (20%)	4 (22.2%)	4 (14.8%)	

Data are n (%) or median with 95% CI. Statistical *p*-value was calculated with: ^a^ Kruskal–Wallis rank sum test, ^b^ Fisher’s exact test. Abbreviations: ALP, alkaline phosphatase; ALT, alanine transaminase; AST, aspartate aminotransferase; Hgb, hemoglobin; ArLD, alcohol-related liver disease; MASH, metabolic dysfunction-associated liver disease; HBV, chronic hepatitis B; HCV, chronic hepatitis C; PBC, primary biliary cholangitis; PSC, primary sclerosing cholangitis; TCMR, T-cell mediated rejection. Under ‘Other’, we included liver conditions such as Fulminant Hepatic Failure (n = 2), Glycogen Storage Disease (n = 1), Hepatopulmonary Syndrome (n = 1), Hepatoma (n = 1), Retransplantation (n = 5).

**Table 2:** Pathway analysis results featuring top altered pathways specific to each two-group comparison.

Group Comparison	Pathway Name	Altered Metabolites from Input List	FDR
**MASH vs. TCMR**	**Alanine, aspartate and glutamate metabolism**	L-Asparagine; Citrate; Fumarate; Succinate; 2-Oxoglutarate	**1.09 × 10^−3^**
	Citrate cycle (TCA cycle)	2-Oxoglutarate; Succinate; Citrate; Fumarate	2.91 × 10^−2^
	Arginine biosynthesis	L-Citrulline; Oxoglutarate; Fumarate	1.46 × 10^−2^
**MASH vs. Biliary**	**Aminoacyl-tRNA biosynthesis**	L-Asparagine; L-Phenylalanine; Glycine; L-Aspartate; L-Valine; L-Alanine; Isoleucine; L-Leucine; L-Tryptophan	**1.35 × 10^−6^**
	Valine, leucine and isoleucine biosynthesis	L-Leucine; L-Isoleucine; L-Valine	2.99 × 10^−3^
	Alanine, aspartate and glutamate metabolism	L-Aspartate; L-Asparagine; L-Alanine; Citrate; Succinate	6.15 × 10^−3^
**Biliary vs. TCMR**	**Butanoate metabolism**	(R)-3-Hydroxybutanoate, Butanoic acid; 2-Oxoglutarate; Succinate	**8.13 × 10^−4^**
	Alanine, aspartate and glutamate metabolism	L-Aspartate; L-Alanine; Succinate; 2-Oxoglutarate	3.75 × 10^−3^
	Arginine biosynthesis	L-Aspartate; L-Citrulline; 2-Oxoglutarate	6.45 × 10^−3^

**Table 3:** Top 20 metabolites identified through ROC curve analysis, showcasing each metabolite’s capacity, as measured by its ROC AUC value, to distinguish between classes.

Metabolite	Biliary	MASH	TCMR
Decanoylcarnitine (C10)	0.811	0.901	0.901
Citric acid	0.832	0.868	0.868
Succinic acid	0.811	0.835	0.835
Phenylalanine	0.779	0.846	0.846
Serine	0.686	0.868	0.868
Dodecanoylcarnitine (C12)	0.739	0.835	0.835
Serotonin	0.812	0.780	0.812
Methylglutarylcarnitine	0.754	0.824	0.824
Hydroxysphingomyeline C22:1	0.832	0.832	0.72
alpha-Aminoadipic acid	0.725	0.824	0.824
Dodecenoylcarnitine (C12:1)	0.700	0.830	0.83
Hexadecenoylcarnitine	0.732	0.808	0.808
Octanoylcarnitine	0.736	0.802	0.802
Alanine	0.779	0.779	0.737
alpha-Ketoglutaric acid	0.710	0.786	0.786
Fumaric acid	0.689	0.791	0.791
Putrescine	0.775	0.775	0.72
Leucine	0.786	0.786	0.687
Sarcosine	0.785	0.686	0.785
Kynurenine	0.689	0.775	0.775

## Data Availability

Metabolomics data., e.g., metabolite concentrations reported in uM units (absolute concentrations) for each sample in this study, is available as Supplementary material (Table S4). Deidentified clinical data used in this project are available by reasonable request to corresponding author, to comply with institutional ethics regulation.

## Supplementary Materials

The following supporting information can be downloaded at: https://www.mdpi.com/article/10.3390/metabo14050254/s1, Supplementary Methods.docx, Figure S1: t-SNE visualization results of samples. A. Projection of the selected metabolites, and B. clinical variables from the original unbalanced dataset. Original samples and those generated using SMOTE. The generated samples closely follow the same distribution as the original dataset. Table S1: PLS-DA VIP scores for top metabolites identified when comparing MASH and TCMR groups. Table S2: PLS-DA VIP scores for top metabolites identified when comparing MASH and Biliary groups. Table S3: PLS-DA VIP scores for top metabolites identified when comparing Biliary and TCMR groups. Table S4: Metabolite concentrations reported in uM units (absolute concentrations) for each sample in this study.
