# Supplementary material for: Harnessing Metabolites as Serum Biomarkers for Liver Graft Pathology Prediction Using Machine Learning
Source: Metabolites. 2024 Apr 27;14(5):254. doi: 10.3390/metabo14050254 (PMC11122840; doi:10.3390/metabo14050254)
Supplement: Supplementary file 1 [file metabolites-14-00254-s001.zip › Figure S1.pdf]

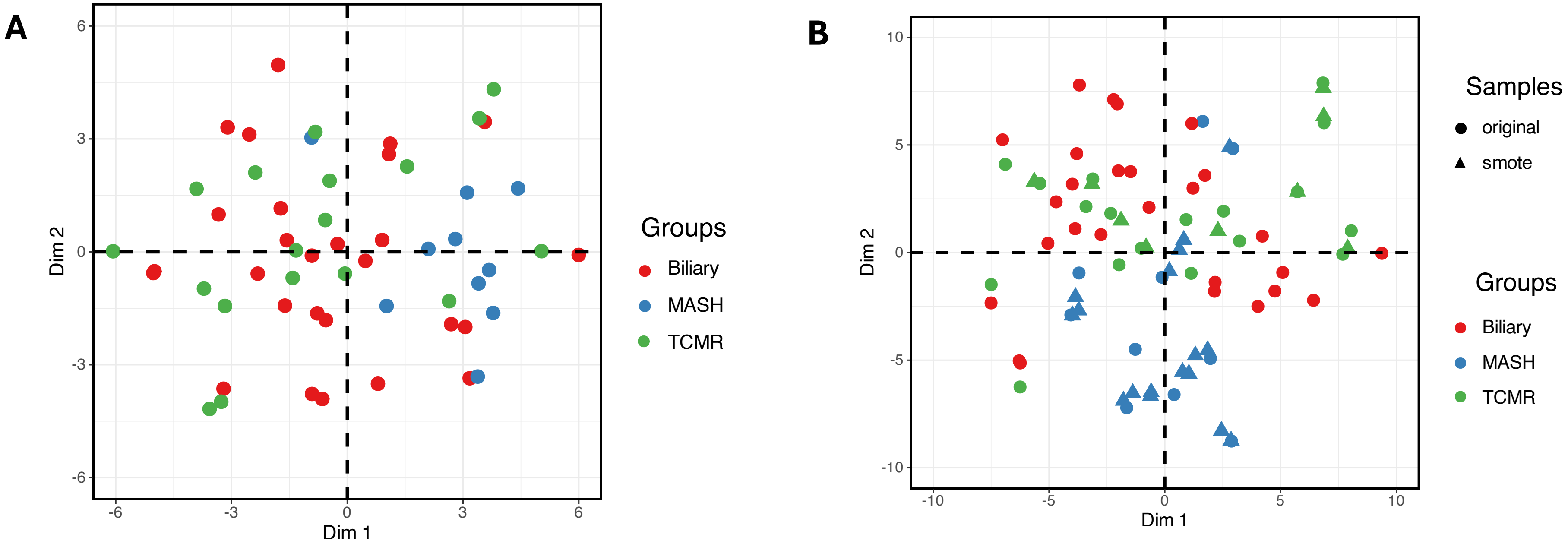

Figure S1: **t-SNE visualization results of samples**. **A**. Projection of the selected metabolites and **B** clinical variables from the original unbalanced dataset. Original samples and those generated using SMOTE. The generated samples closely follow the same distribution as the original dataset.
