# Supplementary material for: Harnessing Metabolites as Serum Biomarkers for Liver Graft Pathology Prediction Using Machine Learning
Source: Metabolites. 2024 Apr 27;14(5):254. doi: 10.3390/metabo14050254 (PMC11122840; doi:10.3390/metabo14050254)
Supplement: Supplementary file 1 [file metabolites-14-00254-s001.zip › Figure S4.pdf]

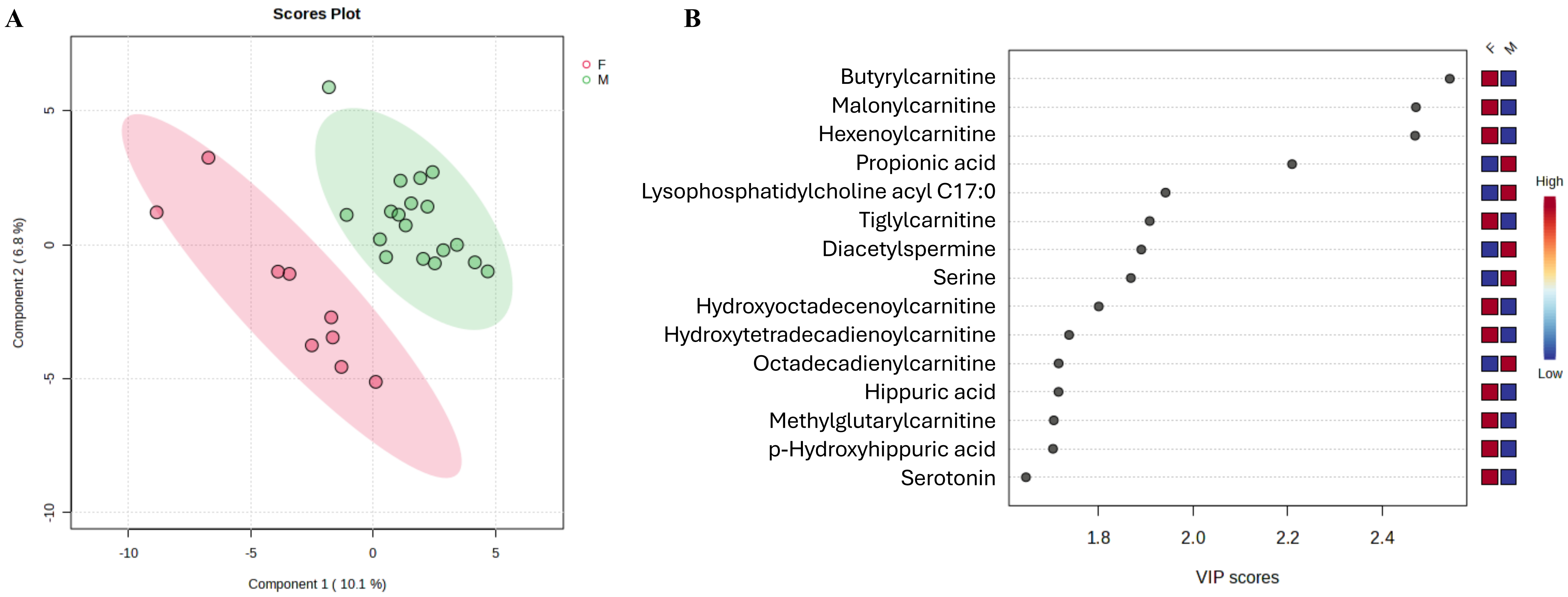

Figure S4. Biliary group – PLS-DA sex-based analysis. **A**. scores plot between the first 2 components and **B** important features identified by PLS-DA.
