# Supplementary material for: Harnessing Metabolites as Serum Biomarkers for Liver Graft Pathology Prediction Using Machine Learning
Source: Metabolites. 2024 Apr 27;14(5):254. doi: 10.3390/metabo14050254 (PMC11122840; doi:10.3390/metabo14050254)
Supplement: Supplementary file 1 [file metabolites-14-00254-s001.zip › Supplementary_Methods.pdf]

Supplementary Methods

An Agilent 1260 series UHPLC system (Palo Alto, CA, U.S.A.) and an Agilent reversed-phase Zorbax Eclipse XDB C18 column (3.0 mm × 100 mm, 3.5 µm particle size, 80 Å pore size) with a Phenomenex (Torrance, CA, U.S.A.) Security Guard C18 pre-column (4.0 mm × 3.0 mm) were used for all online LC/DFI-MS/MS analyses with an AB Sciex QTRAP® 4000 mass spectrometer (Concord, ON, CA). The controlling software for the sample analysis was Analyst® 1.6.2.

The UHPLC parameters used to analyze amino acids and biogenic amines were as follows: solvent A 0.2% (v/v) for-mic acid in water, and solvent B 0.2% (v/v) formic acid in acetonitrile. The gradient profile for this HPLC solvent run was as follows: t = 0 min, 0% B; t = 0.5 min, 0% B; t = 5.5 min, 95% B; t = 6.5 min, 95% B; t = 7.0 min, 0% B; and t = 9.5 min, 0% B. The column oven was set at 50 °C. The flow rate was 500 µL/min, and the sample injection volume was 10 µL. The mass spectrometer was set to a positive electrospray ionization mode with a scheduled multiple reaction monitoring (MRM) scan. The Ion Spray voltage was set at 5500 volts and the temperature at 500 °C. The curtain gas (CUR), ion source gas 1 (GAS1), ion source gas 2 (GAS2) and collision gas (CAD) were set at 20, 40, 50 and medium, respectively.

For DFI-MS/MS analysis, the UHPLC autosampler was connected directly to the MS ion source by red PEEK tubing. The DFI buffer was used as the mobile phase while the flow rate was programmed as follows: t = 0 min, 30 µL/min; t = 1.6 min, 30 µL/min; t = 2.4 min; 200 µL/min; t = 2.8 min, 200 µL/min; and t = 3.0 min, 30 µL/min. The sample injection volume was 20 µL. The mass spectrometer was set to a positive electrospray ionization mode with MRM scanning to analyze lipids and acyl-carnitines, and to a negative electrospray ionization mode to detect glucose/hexose. The Ion Spray voltage was set at 5500 volts for the positive mode and -4500 volts for the negative mode, whereas the temperature was set at 200 °C for both polarities. The CUR, GAS1, GAS2 and CAD were set at 20, 40, 50 and medium, respectively.

For the analysis of organic acids by LC-MS/MS, the solvents used were A) 0.01% (v/v) formic acid in water, and B) 0.01% (v/v) formic acid in methanol. The gradient profile was as follows: t = 0 min, 30% B; t = 2.0 min, 50% B; t = 12.5 min, 95% B; t = 12.51 min, 100% B; t = 13.5 min, 100% B; t = 13.6 min, 30% B and finally maintained at 30% B for 4.4 min. The column oven was set to 40 °C. The flow rate was 300 µL/min, and the sample injection volume was 10 µL. The mass spectrometer was set to a negative electrospray ionization mode with scheduled MRM scanning. The Ion Spray voltage was set at -4500 volts and the temperature at 400 °C. The CUR, GAS1, GAS2 and CAD were set at 20, 30, 30 and medium, separately.
